# Assessment of Epidemiological Trend of Influenza-Like Illness in Italy from 2010/2011 to 2023/2024 Season: Key Points to Optimize Future Vaccination Strategies against Influenza

**DOI:** 10.3390/vaccines12080841

**Published:** 2024-07-25

**Authors:** Sara Boccalini, Angela Bechini

**Affiliations:** Department of Health Sciences, University of Florence, 50134 Florence, Italy; angela.bechini@unifi.it

**Keywords:** epidemiology, children, elderly, older adults, public health, surveillance, ILI, incidence, seasonal influenza

## Abstract

Seasonal influenza is an acute respiratory infectious disease due to influenza viruses, causing a relevant number of illnesses and deaths each year worldwide. Influenza is a preventable disease by vaccination. The aim of this study was to assess the epidemiology of seasonal influenza in Italy through the analysis of data from the epidemiological and virological RespiVirNet surveillance system for the season 2010/2011 to 2023/2024 to identify the epidemiological key points to plan the most appropriate vaccination strategies. The cumulative and maximum weekly incidence of influenza-like illness (ILI) and epidemic period (beginning, end, duration in weeks) were assessed in the pre-pandemic period (2010/2011–2019/2020) and they were compared to the pandemic and post-pandemic one. In all seasons, children reported the highest incidence values of ILI and longer epidemic periods in contrast with the older population. The epidemic seasons 2020/2021 and 2021/2022 had abnormal trends while in the last seasons 2022/2023 and 2023/2024 the epidemiological and virological trends of ILI were confirmed as reported in the pre-pandemic period but with high intensity. Influenza virus A was predominant: the H3N2 subtype circulated more than virus H1N1pdm09. In the few seasons when influenza virus B was the most frequent influenza agent, it co-circulated with influenza virus A. The monitoring of cases is the fundamental tool to better understand the epidemiology of influenza and to optimize future preventive strategies.

## 1. Introduction

Seasonal influenza is an acute infectious disease of the respiratory system caused by influenza viruses, and the disease burden of influenza represents one of the major problems for public health worldwide. Usually, most people recover without requiring medical attention. However, influenza can become a severe illness and sometimes lead to hospitalization and death, particularly among high-risk groups such as younger subjects, older adults, pregnant women, and those with underlying conditions [1].

Worldwide, the annual seasonal influenza affects up to 1 billion people and causes 3–5 million severe cases and up to 650,000 deaths related to respiratory diseases [2]. In Europe, seasonal influenza virus infects approximately 10–30% of the population and causes hundreds of thousands of hospitalizations [3].

Influenza is a preventable disease: each year the influenza vaccination is recommended at specific risk groups to reduce the individual risk of disease, hospitalization, and death, to reduce the risk of virus transmission to subjects at high risk of complications or hospitalization, and, lastly, to reduce the social costs associated with influenza morbidity and mortality [4,5].

Deep and specific knowledge of the epidemiological circulation of the influenza virus is the basic step to managing the immunization program in order to obtain the highest health value. In this scenario, the aim of the current study was to assess the epidemiology of seasonal influenza in the Italian population through the analysis of data collected and published in the periodic reports of the Italian epidemiological and virological surveillance system for the season 2010/2011 to 2023/2024. The main objective is to identify the key points of influenza epidemiology in order to plan the most appropriate and efficient preventive immunological strategies against influenza.

## 2. Materials and Methods

### 2.1. Collection of the Surveillance Data

We collected the epidemiological and virological data registered in the periodic reports of RespiVirNet (https://respivirnet.iss.it/Default.aspx, accessed on 25 July 2024). This is the Italian surveillance system for influenza, which collects the epidemiological and virological data of influenza in the context of the epidemic seasons [6]. It is coordinated by the Istituto Superiore di Sanità (ISS) in collaboration with the Italian Ministry of Health. Sentinel doctors report weekly cases of influenza-like illness (ILI) observed among their patients and collect biological samples for the identification of circulating influenza viruses. The ISS collects and processes ILI and laboratory data to prepare and publish the weekly epidemiological and virological bulletins. Surveillance is active from the 42nd week of the year until the 17th week of the following year [6]. ILI epidemiological data are used as a proxy for influenza incidence. Epidemiological and virological surveillance bulletins published in the period from 2010/2011 to 2023/2024 seasons were collected and the reported data were analyzed.

### 2.2. Epidemiological Surveillance Data

An analysis of the epidemiological data of influenza seasons of interest, for the total population and for different age groups (subjects 0–4 years, 5–14 years, 15–64 years, ≥65 years old), was performed by evaluating the following indicators for each season:

- Cumulative incidence of ILI;
- Maximum weekly incidence of ILI;
- Epidemic period (beginning, end, duration in weeks).

Average values of the above data were calculated for the COVID-19 pre-pandemic period from 2010/2011 to 2019/2020. The following influenza seasons have been excluded from those calculations because they are strongly influenced by the circulation of SARS-CoV-2 and its variants and other etiological agents and by the adoption of pandemic control interventions. The epidemiological data of the pre-pandemic period were compared to those referring to the pandemic and following periods (as a matter of fact, the last two seasons, 2022/2023 and 2023/2024).

To have a homogeneity in the definition of the epidemic period for all the influenza seasons examined, a weekly incidence value of ≥2 cases of ILI for 1000 people assisted was considered as a baseline level for the beginning of the seasonal epidemic of influenza.

### 2.3. Virological Surveillance

An assessment of the virological data of influenza virus circulation in the epidemic seasons of interest from 2010/2011 to 2023/2024 has also been carried out. In particular, we collected data on the number of analyzed samples and the number of positive samples of influenza virus and influenza virus A (H1N1pdm09 and H3N2) and B. Therefore, the annual rate of positive samples for influenza virus on the total number of samples collected and analyzed in the laboratories in each season has been determined. In addition, an assessment of the virological distribution of influenza A and influenza B viruses among the positive samples was conducted in order to value the most frequent influenza virus in the different seasons. Lastly, an analysis of the typing of samples positive for influenza A viruses (H1N1pdm09, H3N2, A virus not subtyped) was performed. The influenza B viruses were not constantly typed over time in Italy and, therefore, were not included in the current analysis.

## 3. Results

### 3.1. Epidemiology of Seasonal Influenza in Italy

In Italy in the 10 pre-pandemic seasons observed from 2010/2011 to 2019/2020, 10.4% of the general population was affected on average by ILI (Table 1). The 2017/2018 season showed a very high level of epidemic intensity, with the highest cumulative incidence values of ILI for all age groups compared to other seasons and an average incidence value of 14.3% for the total population. On the other hand, the 2013/2014 season had the lower cumulative incidence (7.6%). The influenza 2020/2021 season shows an “abnormal” trend of ILI compared to previous seasons, with an incidence value for ILI at a historic minimum (4.0%) in all age groups due to the adoption of pandemic control interventions. The 2021/2022 epidemic season showed a new increase in the cumulative incidence of ILI up to 11.0% in the general population compared to the previous season. In the last two seasons (2022/2023 and 2023/2024), the cumulative incidence of ILI was very high in all age groups, with values more than double that of the previous period (23.7% and 24.8%, respectively) (Table 1).

In the pre-pandemic period, the pediatric and adolescent populations (subjects 0–4 years and 5–14 years old) were characterized by higher cumulative incidence rates of ILI (on average, 26.8% and 16.8%, respectively) in all observed seasons. On the other hand, the older population showed lower cumulative incidence values of ILI than the other younger age groups in all influenza seasons, declining up to 4.4% (on average) in the older adults. This decreasing trend of incidence when the age increases was also reported in the pandemic and post-pandemic period. Particularly, the high intensity of ILI in the 2022/2023 season was described in all age groups with values more than doubled compared to the average data in the 10-year pre-pandemic period. A slightly lower intensity than the 2022/2023 period was reported in the last 2023/2024 season in younger population and an opposite trend in older subjects (Table 1).

**Figure 1:**
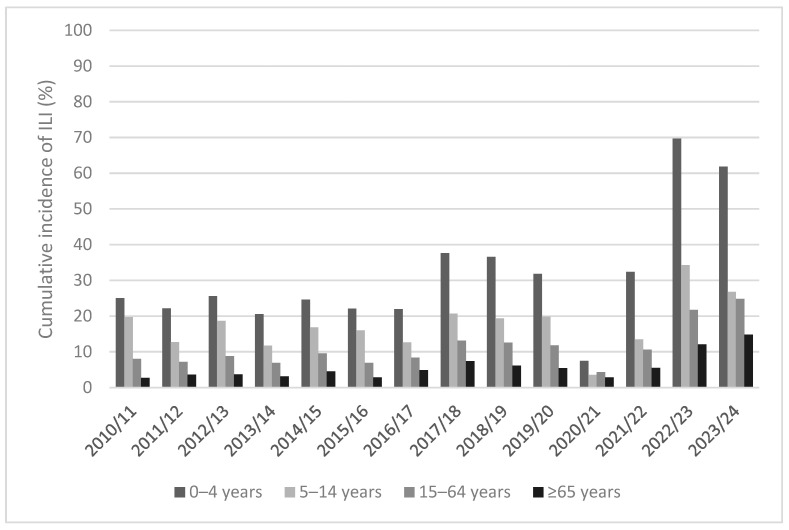
Cumulative incidence of ILI (%) from 2010/2011 to 2023/2024 season by age groups.

Particularly, in the pre-pandemic period, during the 2017/2018 and 2013/2014 seasons, the higher and the lower annual cumulative incidence rates of ILI were reached in all age groups, respectively (Figure 1). However, in the abnormal 2020/2021 season the cumulative incidence was almost halved in all age groups compared to the lowest-intensity season 2013/2014 of the pre-pandemic period. In the 2021/2022 season, with an increase in the ILI cases in all age groups compared to the previous 2020/2021 season (which had had an basal intensity), the cumulative incidence of ILI by age groups was close to average pre-pandemic values. In the last seasons 2022/2023 and 2023/2024, the cumulative incidence of ILI almost doubled in all age groups, as in the general population (Figure 1).

The trend of the weekly incidence of ILI in the influenza seasons from 2010/2011 to 2023/24 is comparable with that of the cumulative incidence. The highest value of weekly incidence of ILI (on average) reported in the pre-pandemic period was 10.5 cases per 1000 patients in the general population (Table 2). In the 2017/2018 and 2015/2016 seasons, the higher and lower weekly incidence values (14.7‰ and 6.1‰) in the total population were recorded, respectively. However, in the pandemic period, as expected, the weekly incidence value reached a lower value (2.0‰). The 2021/2022 season shows two abnormal epidemic peaks, instead of one, with a maximum weekly incidence of 5.2 cases for 1000 patients in December (Week 52) and 5.3 cases for 1000 patients in March–April (Week 13). The 2022/2023 season reports the typical epidemiological trend of ILI with a maximum weekly incidence of 15.7‰ in the general population (value 1.5 times higher than the average value of the previous pre-pandemic period). However, the maximum weekly incidence of ILI (18.4‰) was notified in the last 2023/2024 season (Table 2).

**Figure 2:**
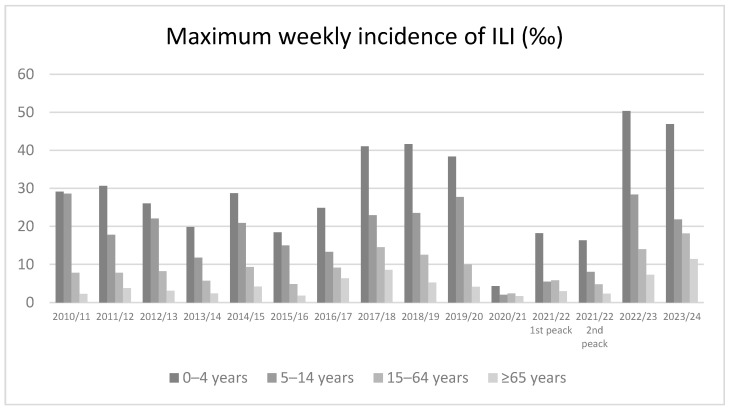
Maximum weekly incidence of ILI (%) from 2010/2011 to 2023/2024 season by age groups.

In particular, as reported for the trend of the cumulative incidence, in the pre-pandemic period the value of maximum weekly incidence of ILI by age groups was higher in the pediatric population (29.9‰) in all observed seasons and it decreased in all other older age groups up to 4.2‰ in the adults ≥65 years of age (on average) (Table 2). This trend by age group is reported in each season. Particularly, in the pre-pandemic period the maximum weekly incidence of ILI achieved a value of 41.6 cases per 1000 children in the 2018/2019 season. After the relevant reduction reported in the pandemic 2020/2021 season, the values of maximum weekly ILI incidence increased in the 2021/2022 season in all age groups and reached higher values in the period 2022/23 compared to all previous seasons (almost twice that of the pre-pandemic period). Particularly, the values of maximum weekly incidence of ILI were 50.4% and 28.4 ‰ in subjects 0–4 and 5–14 years old. In the last 2023/2024 season, the maximum weekly incidence was lower in the 0–14 years group and higher in the ≥15 years group compared to the previous season (Figure 2).

**Figure 3:**
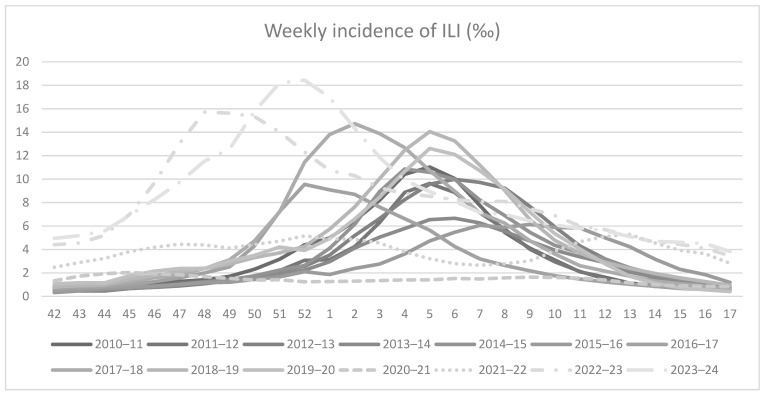
Weekly incidence of ILI (%) from 2010/2011 to 2023/2024 seasons.

Generally, the maximum weekly incidence of ILI occurs around the 5th week of the year (end of January–early February) in the pre-pandemic period, although in some seasons the epidemic peak was reached earlier (52th week in the 2016/2017 season) or later (8th week in the 2015–2016 season) (Figure 3).

There are no relevant differences among age groups, even if usually the peaks were reported slightly before in the younger groups. In the 2020/2021 season, the maximum weekly incidence of ILI was particularly anticipated (45th week) even if it was very limited. In the 2021/2022 season, two peaks of weekly incidence of ILI were reached in the 5th and 13th weeks, respectively. An anticipated top weekly incidence of ILI was also reported in the 2022/2023 season (48th week) while in the last 2023/2024 season the higher incidence was reached in the 52th week (Table 3).

Considering a weekly incidence of two cases of ILI per 1000 patients as the epidemic threshold, on average the epidemic period begins around the 50th week (2nd week of December), finishes around the 12th week of each year (the 3rd week of March), and has a duration of 16 weeks in the general population during the 10 seasons from 2010/2011 to 2019/2020 (Table 4). In the pre-pandemic period, the seasons 2018/2019 and 2019/2020 were characterized by an early beginning and a longer duration of the epidemic period (19 weeks) while in the 2011/2012 season, the epidemic period was shorter (13 weeks). For the 2020/2021 season, only in correspondence to the 45th week of surveillance, the weekly incidence value of ILI was over 2 cases for 1000 patients in the total population. In the last 3 seasons (from 2021/2022 to 2023/2024), the weekly incidence was over 2 cases of ILI per 1000 patients for the entire surveillance period (from the 42nd week to the 17th week); however, in the 2021/2022 season, the incidence of ILI was low. Meanwhile, in the 2022/2023 and 2023/2024 seasons, it was very high (Table 4).

The analysis of the epidemic period by age groups for the 2010/2011–2019/2020 seasons highlights how usually the onset of the season is anticipated in the pediatric population (45th week) compared to older age groups (Table 5). In particular, the start of the epidemic period is postponed in the older population (the first week of the year). In parallel, the end of the epidemic period takes place later for the population of the first years of life (on average towards the 15th week for subjects aged 0–4 years) compared to other age groups. In fact, the end of the epidemic period occurs earlier in other age groups, for example, around the 8th week for subjects older than 65 years. This determines that on average the epidemic period is more prolonged in the age group 0–4 years (24 weeks) and is definitely more reduced in the older population (about 9 weeks). Particularly, in the 2015/2016 season the ILI cases were always under the threshold of 2 cases per 1000 patients in subjects ≥65 years of age. The same situation was reported in the 2020/2021 season. In the last three seasons, the weekly incidence of ILI was almost always over 2 cases of ILI per 1000 patients in all age groups for the entire surveillance period, with the exception of subjects ≥65 years of age. Particularly, in the 2021/2022 season the first epidemic period lasted 10 weeks (from Week 46 to Week 3) while the second one lasted 3 weeks (from Week 42 to Week 44) in older adults. In the last 2022/2023 and 2023/2024 seasons, the weekly incidence of ILI was over 2 cases of ILI per 1000 older subjects for 26 weeks and 27 weeks, respectively, out of 28 weeks of surveillance (Table 5).

### 3.2. Virological Surveillance of Influenza

In the pre-pandemic period, the average rate of positivity for one of the influenza viruses (A virus or B virus) of the biological samples examined by the Italian virological surveillance system was 31%. The highest positive rate (38%) was reported in the 2012/2013 season. Also, for the seasons 2011/2012, 2014/2015, and 2017/2018 high positive rates were obtained (about 35%). In the seasons before the pandemic period, the minimum positive rate, equal to 23%, was recorded in the seasons 2013/14 and 2019/2020. However, data for the 2019/2020 season may have been affected by the circulation of SARS-CoV-2, the related restriction measures taken from the end of February/beginning of March 2020, and the reduction in the activity of the virological surveillance system of influenza due to the pandemic emergency. In the season 2020/2021, there was a total absence of influenza viruses in the collected biological samples, facing an extremely small number of samples taken (about 6800). The 2021/2022 season was characterized by a very low rate of positivity (15%) due to the circulation of other pathogens. Particularly, in that season, SARS-CoV-2 was identified in 29% of samples. In the season 2022/2023, the influenza-positive rate grew to 22%: SARS-CoV-2 was identified in 6.3% of samples while 21.9% of samples were positive to other respiratory viruses (mainly RSV with a positivity rate of 11%). In the 2023/2024 season, the positive rate was 13%: influenza virus was identified in 41% of total samples, followed by SARS-CoV-2 (17%), RSV (16%), and other respiratory pathogens (Table 6).

**Figure 4:**
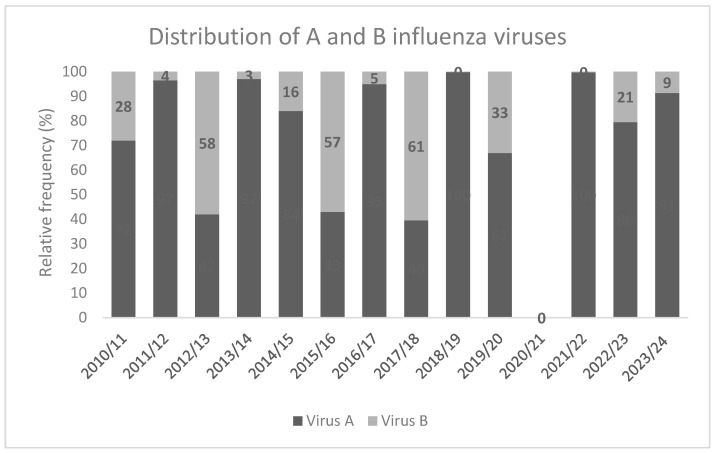
Distribution of A and B influenza viruses from 2010/2011 to 2023/0224 season.

In the 10-year period 2010/2011–2019/2020, influenza A viruses were more frequently identified compared to influenza B viruses (Figure 4). In particular, in four seasons (2011/2012, 2013/2014, 2016/2017, and 2018/2019) influenza A viruses accounted for almost all viruses isolated from positive samples (more than 95%). However, even in the other three seasons (2010/2011, 2014/2015, 2019/2020) the frequency of A viruses was very high, ranging from 67% to 85%. Instead, B influenza viruses circulated more frequently during the 2012/2013 (58%), 2015/2016 (57%), and 2017/2018 (61%) seasons. It should be stressed that, even when B viruses were prevalent, they were isolated in up to 60% of the positive samples. In the 2021/2022 season, almost only influenza virus A (99.7%) was reported. The high prevalence of influenza virus A circulation (79.5% and 91.3%) was confirmed also in the last seasons 2022/2023 and 2023/2024 (Figure 4).

**Figure 5:**
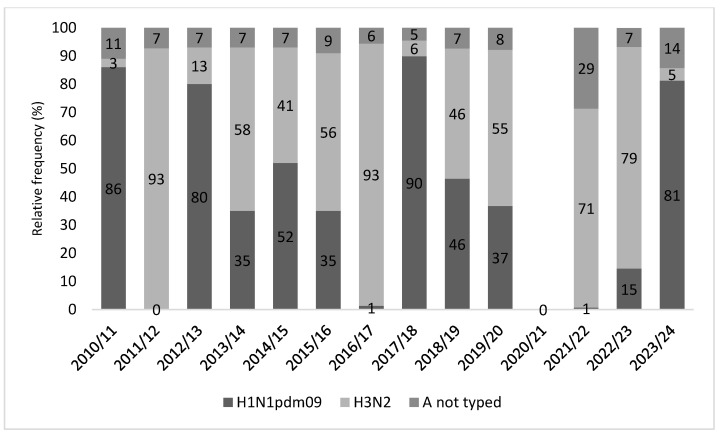
Distribution of influenza A viruses from 2010/2011 to 2023/24 season.

With regard to the influenza A viruses, there is some variability in frequency between the subtype H1N1pdm09 and the subtype H3N2 in the observed period. The subtype H1N1pdm09 was more frequent in 3 seasons (2010/2011, 2012/2013, and 2017/2018) with high rates (86%, 80%, and 89.9%, respectively) in the pre-pandemic period. Instead, during two seasons (2011/2012 and 2016/2017) almost all viruses A isolated belonged to the subtype H3N2. Finally, in the other five seasons, H1N1pdm09 and H3N2 virus co-circulated. In the 2021/2022 season, the subtype H3N2 was isolated in 71% of positive samples (note that 28.7% of samples have not been typed). A similar frequency was also reported in 2022/2023. Instead, in the last season 2023/2024, the subtype H1N1pdm09 was the most frequently circulating influenza virus A (Figure 5).

## 4. Discussion

The aim of this study was to assess the epidemiological and virological trends of the annual influenza epidemics in Italy from the 2010/2011 season up to now in order to plan the most appropriate preventive strategies in the future.

Time patterns of ILI cases reported in the Italian surveillance system show a relevant variability in the annual epidemic trends, with seasons when the intensity of incidence was very low and others when the intensity was very high, as in the last seasons before the pandemic period and the last seasons. However, some key points are evident from the analysis of the epidemiological surveillance data.

The pediatric and adolescent populations have always reported the highest incidence values of ILI and a longer epidemic period. In particular, in the age group 0–4 years, the epidemic period on average begins at Week 45 of each year (the second week of November), and extends for almost the entire influenza season, with an average duration of 24 weeks. In addition, the average cumulative incidence in this age group (27%) in the pre-pandemic period highlights that more than one-quarter of children (about 558,000 subjects) had ILI each year. This value grew at >60% in the last influenza season. Particularly, considering about 2,000,000 children 0–4 years old in Italy [7], about >60,000 children have ILI on average in the week with the maximum value of weekly incidence. In the last seasons >100,000 children had ILI in the week of incidence peak. Even if influenza has generally a benign course in these age groups, however, this high number of subjects with ILI has a relevant impact on the health care systems and society. As a matter of fact, influenza may be responsible for hospital admissions (especially in younger children) and for a significant number of lost school days, lost work days by parents, and increased consumption of health resources due to medical examinations, use of antipyretic drugs and use of antibiotics [8]. In addition, it should be noted that, due to the high incidence rates and the anticipated outset of the epidemic period in this age group compared with the others, the younger population is the main source of infection for the general population and, in particular, can transmit the infection to categories of subjects most at risk of severe morbidity and mortality related to influenza syndrome such as older adults or subjects with chronic disease (diabetics, pulmonary disease, heart disease, etc.) [9].

Epidemiological surveillance data, however, show a different trend for older adults. The older population is characterized by the lowest rates of ILI incidence (cumulative and maximum week incidence) and a reduced epidemic period (8 weeks on average), with a start towards the beginning of the year (Week 1) and a conclusion at the end of February (Week 8). More than 4% of older adults had ILI each year in the pre-pandemic period but this rate corresponds to >600,000 subjects (considering an older population of 14,000,000 subjects [7]) and this value has tripled in the last 2 influenza seasons (12% and 15%, respectively). Particularly, about 60,000 subjects had ILI in the week of maximum weekly incidence on average in the pre-pandemic period and this number increased over the years. Therefore, even if the incidence rate is low, the number of cases is consistent. As a matter of fact, it is appropriate to emphasize how the subjects in this age group are at increased risk for influenza complications (which may also evolve into outcomes particularly negative, such as death) due the age and other co-morbidities [10], leading to a high disease burden with a relevant health and societal impact.

The last weeks of the 2019/2020 influenza season were affected by the circulation of SARS-CoV-2 [6]. From surveillance data, it seems that the seasonal epidemic stopped abruptly in advance to the 13th week of 2020. Probably, the prevention and protection measures taken in response to the pandemic emergency could have blocked regular and continuous surveillance or slowed the circulation of influenza viruses in the population [11]. The 2020/2021 influenza season had an abnormal trend compared to previous seasons, with extremely low ILI incidence rates and a total absence of influenza viruses identified in respiratory samples collected and analyzed by the virological surveillance system. Globally, also in the temperate regions of the northern hemisphere, there was an analogous trend, with an influenza season characterized by a viral circulation well below inter-seasonal levels, with sporadic isolations of viruses A and B [6].

In the 2021/2022 season, a resumption of intensity was observed (although at low values) of the influenza season (in terms of maximum weekly and cumulative ILI incidence) compared to the previous season 2020/2021 at the Italian level. In particular, the 2021/2022 season was characterized by two unusual epidemic peaks: a first epidemic peak between Week 44 and Week 5 and a second peak that extended between Week 10 and 17; the season ended with an ILI incidence level of 2.83 cases per 1000 patients. In the 2019/2020 season, the last season with an epidemic of ILI before the pandemic, the estimated incidence of ILI in the 17th week (last week of surveillance) was much less (0.42‰). In addition, the rate of positivity of the biological samples analyzed by the virological surveillance system was very low (15%), almost half the average calculated value for influenza seasons from 2010/2011 to 2019–2020, indicating the co-circulation of other pathogens, including the SARS-CoV-2 (identified in 29% of samples). Apart from these issues, the key points highlighted in the previous 10-year period before the SARS-CoV-2 pandemic continued to be evident. Therefore, after the significant reduction in the incidence of ILI in the pandemic emergency period 2020/2021 due to control and prevention of infection by SARS-CoV-2, the data of the 2021/2022 season show that influenza viruses continue to circulate, and their spread is increased as the non-pharmacological restrictions applied for the control of the pandemic of COVID-19 have been reduced. This issue is confirmed by the high intensity notified in the last seasons 2022/2023 and 2023/2024, when the epidemiological and virological trends of ILI were confirmed as reported in the last decades but with high intensity.

Another key point is that in the years the start of the epidemic period has been increasingly anticipated. This issue should be taken into account in the planning of the annual vaccination program.

Moreover, the rate of positive samples of the influenza virus, accounting for 31% of the pre-pandemic virus, decreased in the last years despite the fact that the number of samples has significantly increased. It could be related to the great attention to respiratory viruses after the pandemic and the consequent strengthening of the ILI monitoring system. Analysis of virological surveillance data shows that in the analyzed influenza seasons there is a predominant circulation of influenza virus A. Among the viruses A, the H3N2 subtype circulated more than virus H1N1pdm09 in the observed period. In the few pre-pandemic seasons when influenza virus B was the most frequent influenza agent, it co-circulated with influenza virus A and the percentage of influenza virus B did not exceed 60%. Influenza virus B disappeared in the pandemic period (2020/2021 and 2021/2022 seasons) and later it had a limited distribution (21% and 9% in 2022/2023 and 2023/2024 seasons, respectively) due to B/Victoria lineage virus. As a matter of fact, globally, there have been no confirmed detections of circulating B/Yamagata lineage viruses after March 2020 [12]. In the last few years, the co-circulation of other respiratory pathogens become more evident.

The main limitation of this study is that we assess epidemiological and virological data collected by the surveillance system of ILI and not of laboratory influenza-confirmed cases. However, the ILI can be considered an optimal proxy of influenza case trends, even when there is the circulation of other respiratory agents, as in the last seasons.

This study did not assess any relationship between epidemic season intensity and vaccination coverage in the general population and older adults. The current Italian National Immunization Plan 2023–2025 and the annual Ministerial Circulars for the Prevention and Control of Influenza have set the minimum achievable vaccination coverage goal at 75% and the optimal one at 95% for subjects over sixty-five years of life and high-risk groups [4,13]. However, the influenza vaccine coverage in older adults ranged from 62.4% to 48.6% from the 2010/2011 to 2019/2020 seasons. The vaccine coverage increased in 2020/2021 (65.3%) and decreased in the two seasons 2021/2022 and 2022/2023 (58.1% and 56.7%, respectively) in this age group. Instead, the vaccine coverage of the general population was always <24% [14]. On the other hand, to our knowledge, this is the first study that summarized epidemiological and virological data collected in more than 10 years of surveillance, highlighting some useful key points to consider in future preventive strategies. For example, even if influenza is usually benign in children, the younger population has the highest influenza incidence and the earliest outset of epidemic season. These data confirm the value of vaccinating children and paying attention to beginning the immunization program as soon as possible. This issue could be useful to reduce the number of cases in younger people but also to reduce the virus transmission to older subjects (who usually begin the epidemic season later). Concerning the older adults, they have a lower influenza incidence; however, considering the demographic structure of the Italian population, the number of influenza cases is very relevant in this already fragile population. It has a relevant impact on our healthcare systems each year. In addition, a great number of older adults have other underlying conditions that increase the risk of complications and death.

## 5. Conclusions

Influenza represented and will continue to represent one of the major problems of Public Health due to the high incidence of cases notified each year. The careful epidemiological and virological monitoring of cases is the fundamental tool to better understand and predict the epidemiology of influenza in the future and, in the end, optimize preventive strategies for the Italian population.

## Figures and Tables

**Table 1:** Cumulative incidence of ILI (%) from 2010/2011 to 2023/2024 season by age groups.

Cumulative Incidence of ILI (%)					
Season	0–4 Years	5–14 Years	15–64 Years	≥65 Years	Total Population
2010/2011	25.1	19.7	8.0	2.7	9.8
2011/2012	22.2	12.7	7.2	3.7	8.2
2012/2013	25.6	18.7	8.8	3.7	10.2
2013/2014	20.6	11.8	6.9	3.1	7.6
2014/2015	24.6	16.9	9.6	4.5	10.4
2015/2016	22.1	16.0	6.9	2.8	8.0
2016/2017	22.0	12.6	8.4	4.9	9.0
2017/2018	37.6	20.7	13.2	7.4	14.3
2018/2019	36.6	19.3	12.6	6.1	13.4
2019/2020	31.8	19.9	11.8	5.5	12.6
**Average for the period 2010/2011–2019/2020**	**26.8**	**16.8**	**9.3**	**4.4**	**10.4**
2020/2021	7.5	3.5	4.3	2.8	4.0
2021/2022	32.4	13.5	10.6	5.5	11.0
2022/2023	69.7	34.3	21.8	12.1	23.7
2023/2024	61.8	26.8	24.8	14.8	24.8

**Table 2:** Maximum weekly incidence of ILI (‰) from 2010/2011 to 2023/2024 season by age groups.

Maximum Weekly Incidence of ILI (‰)					
Season	0–4 Years	5–14 Years	15–64 Years	≥65 Years	Total Population
2010/2011	29.1	28.6	7.8	2.3	11.0
2011/2012	30.6	17.8	7.8	3.8	9.6
2012/2013	26.1	22.1	8.2	3.1	10.0
2013/2014	19.8	11.8	5.7	2.4	6.7
2014/2015	28.7	20.9	9.3	4.2	10.9
2015/2016	18.4	15.0	4.8	1.8	6.1
2016/2017	24.9	13.3	9.2	6.3	9.6
2017/2018	41.0	23.0	14.5	8.6	14.7
2018/2019	41.6	23.5	12.5	5.2	14.1
2019/2020	38.3	27.7	10.0	4.1	12.6
**Average for the period 2010/2011–2019/2020**	**29.9**	**20.4**	**9.0**	**4.2**	**10.5**
2020/2021	4.3	2.0	2.4	1.7	2.0
2021/2022 1st peak	18.2	5.5	5.8	3.0	5.2
2021/2022 2nd peak	16.4	8.0	4.8	2.3	5.3
2022/2023	50.4	28.4	14.0	7.3	15.7
2023/2024	46.9	21.8	18.1	11.4	18.4

**Table 3:** Week of maximum weekly incidence of ILI from 2010/2011 to 2023/2024 season by age groups.

Week of Maximum Weekly Incidence of ILI					
Season	0–4 Years	5–14 Years	15–64 Years	≥65 Years	Total Population
2010/2011	5	5	5	2	5
2011/2012	4	4	5	5	5
2012/2013	6	5	7	7	6
2013/2014	5	6	6	7	6
2014/2015	4	4	4	6	4
2015/2016	8	8	6	8	8
2016/2017	52	51	1	1	52
2017/2018	4	3	2	1	2
2018/2019	5	5	5	5	5
2019/2020	5	5	5	5	5
**Average for the period 2010/2011–2019/2020**	**5**	**4**	**5**	**5**	**5**
2020/2021	8	5	45	46	45
2021/2022 1st peak	46	47	52	52	52
2021/2022 2nd peak	13	13	13	13	13
2022/2023	49	48	50	50	48
2023/2024	51	51	1	52	52

**Table 4:** Week of outset and end of the epidemic season of ILI, and duration of the epidemic period from the 2010/2011 to 2023/2024 seasons in the total population.

Epidemic Period in the Total Population			
Season	Outset of Epidemic Period (Week)	End of Epidemic Period (Week)	Duration (Number of Weeks)
2010/2011	50	11	14
2011/2012	51	11	13
2012/2013	51	13	15
2013/2014	52	13	14
2014/2015	51	13	15
2015/2016	52	14	16
2016/2017	48	9	14
2017/2018	48	12	17
2018/2019	47	13	19
2019/2020	46	12	19
**Average for the period 2010/2011–2019/2020**	**50**	**12**	**16**
2020/2021	45	45	1
2021/2022	42	17	28
2022/2023	42	17	28
2023/2024	42	17	28

**Table 5:** Average of the week of outset and end of the epidemic season, and duration of the epidemic period from the 2010/2011 to 2019/2020 seasons (pre-pandemic period) by age groups.

Averages for the Period 2010/2011–2019/2020	0–4 Years	5–14 Years	15–64 Years	≥65 Years	Total Population
Outset of epidemic period (week)	45	49	45	1	50
End of epidemic period (week)	15	13	12	8	12
Duration of epidemic period (weeks)	24	18	15	9	16

**Table 6:** Number of samples collected, number of influenza-positive samples and rate of positivity of the samples analyzed on the total collected.

Season	Total Number of Samples Collected	Total Number of Positive Samples	Positive Rate
2010/2011	9229	2880	31%
2011/2012	4667	1671	36%
2012/2013	5535	2125	38%
2013/2014	4444	1036	23%
2014/2015	10,299	3708	36%
2015/2016	8971	2422	27%
2016/2017	12,034	3518	29%
2017/2018	16,135	5494	34%
2018/2019	20,009	6368	32%
2019/2020	16,146	3760	23%
**Average 2010/2011–2019/2020**	**10,747**	**3298**	**31%**
2020/2021	6818	0	0%
2021/2022	13,063	1899	15%
2022/2023	28,977	6325	22%
2023/2024	59,662	7985	13%

## Data Availability

The original contributions presented in the study are included in the article, further inquiries can be directed to the corresponding author.
